# Sphingosine‐1‐phosphate receptor 3 is implicated in BBB injury via the CCL2‐CCR2 axis following acute intracerebral hemorrhage

**DOI:** 10.1111/cns.13626

**Published:** 2021-02-28

**Authors:** Dingkang Xu, Qiang Gao, Fang Wang, Qianrui Peng, Guoqing Wang, Qingjie Wei, Shixiong Lei, Shengqi Zhao, Longxiao Zhang, Fuyou Guo

**Affiliations:** ^1^ Department of Neurosurgery the First Affiliated Hospital of Zhengzhou University Zhengzhou Henan PR China; ^2^ Henan Key Laboratory of Child Brain Injury Institute of Neuroscience and Third Affiliated Hospital Zhengzhou University Zhengzhou Henan PR China

**Keywords:** blood‐brain barrier, intracerebral hemorrhage, P38 MAPK pathway, secondary brain injury, sphingosine‐1‐phosphate receptor 3

## Abstract

**Background:**

Intracerebral hemorrhage (ICH) is a catastrophic cerebrovascular disease with high morbidity and mortality. Evidence demonstrated that sphingosine‐1‐phosphate receptor (S1PR) plays a vital role in inflammatory damage via the upregulation of CCL2 expression. However, whether S1PR3 is involved in blood‐brain barrier (BBB) breakdown via CCL2 activation after ICH has not been described.

**Methods:**

We investigated the expression profiles of all S1PRs using high‐throughput RNA‐seq analysis and RT‐PCR. The potential role of S1PR3 and interaction between S1PR3 and CCL2 were evaluated via Western blotting, immunofluorescence, and flow cytometry. BBB disruption was examined via magnetic resonance imaging, transmission electron microscopy, and Evans blue extravasation. Microglial activation, proliferation, and polarization were assessed via histopathological analysis. The expression levels of CCL2, p‐p38 MAPK, ICAM‐1, and ZO‐1 were examined in vitro and in vivo.

**Results:**

The present results showed that the levels of S1PR3 and its ligand, sphingosine 1‐phosphate (S1P), were dramatically increased following ICH, which regulated the expression of CCL2 and p38MAPK. Moreover, reductions in brain edema volume, amelioration of BBB integrity, and improvements in behavioral deficits were achieved after the administration of CAY10444, an S1PR3 antagonist, to rats. Remarkably increased CCL2, p‐p38MAPK, and ICAM‐1 expression and decreased ZO‐1 expression were observed in cocultured human astrocytes (HAs) and hCMEC/D3 cells after S1P stimulation. However, the expression levels of CCL2, p‐p38 MAPK, and ICAM‐1 were decreased and ZO‐1 expression was increased after S1PR3 inhibition. In addition, microglial proliferation and M1 polarization were attenuated after CAY10444 administration.

**Conclusion:**

To the best of our knowledge, this is the first demonstration of the neuroprotective role of S1PR3 modulation in maintaining BBB integrity by inhibiting the S1PR3‐CCL2 axis after ICH, providing a novel treatment for ICH by targeting S1PR3.

## Graphical abstract

CAY10444 could alleviate early inflammation response after ICH and exert neuroprotective effect via the S1P‐CCL2‐p‐p38 MAPK pathway. Microglial proliferation and M1 polarization were attenuated by S1PR3 blockage.

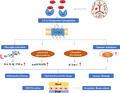

**Abbreviations**
BBB: Blood‐brain barrier
BMEC: Brain microvascular endothelial cell
CCL2: Chemokine CC ligand 2
ICAM‐1: Intercellular cell adhesion molecule‐1
ICH: Intracerebral hemorrhage
p‐p38MAPK: Phosphorylated p38 mitogen‐activated protein kinase
S1P: Sphingosine‐1‐phosphate
ZO‐1: Zonula occludens‐1

## 1 INTRODUCTION

Intracerebral hemorrhage (ICH) is a devastating cerebrovascular disease that accounts for 10%‐15% of all stroke events.^1^, ^2^, ^3^ Numerous efforts were made in clinical studies over the past few years, but no currently available medical treatments provide sufficient benefits to patient outcome or neurological function.^4^, ^5^, ^6^ Mounting evidence suggests that excessive inflammation triggers the mechanism underlying secondary brain injury after ICH.^7^, ^8^, ^9^, ^10^ Our previous study demonstrated that the chemokine CCL2, its receptor CCR2, and the p38 MAPK pathway contribute to blood‐brain barrier (BBB) disruption and brain edema.^11^ However, whether an existing upstream molecule modulates CCL2 in BBB breakdown was not clearly defined.

The BBB is a highly specialized system for regulating the passage of substances from peripheral fluids into the brain, which leads to the maintenance of brain homeostasis. However, marked disruption of the BBB is observed following ICH, accompanied by neuroinflammatory responses and vasogenic edema formation. Previous research identified that sphingosine‐1‐phosphate (S1P) and S1P receptor (S1PR) were involved in impaired BBB function in various central nervous system (CNS) diseases.^12^, ^13^, ^14^ S1P is a bioactive lipid that exerts various pathophysiological functions via binding to its receptor family of G protein‐coupled receptors, and it is extremely important to identify which of the five identified S1PR subtypes is involved in ICH.^12^ Accumulating evidence demonstrated that S1PR3 influenced proinflammatory M1 polarization and activated the p38 MAPK pathway that is involved in the brain injury following ischemic stroke, and astrocytic S1PR3 modulated the blood‐tumor barrier via regulation of CCL2 secretion in brain metastases.^15^ Fingolimod is a non‐selective S1PR antagonist that attenuated neurological deficits by preventing lymphocyte egress from lymphoid organs in a rodent model of ICH,^16^ and it entered clinical trials for the treatment of ICH.^17^ However, whether S1PR3 is implicated in BBB breakdown via CCL2 regulation after ICH is not known.

The present study assessed the possible role of the S1P‐S1PR signaling pathway in hemorrhagic stroke and focused on changes in the features of S1PR within the brain. The potential mechanisms of a specific S1PR subtype in BBB disruption and brain edema was investigated in acute ICH. Our data suggest S1PR3 as a possible therapeutic target for ICH.

## 2 METHODS

### 2.1 Patient selection

Five acute ICH samples and five normal brain tissue samples were collected from the First Affiliated Hospital of Zhengzhou University, PR China. The Ethics Committee for Human Experiments of Zhengzhou University approved all procedures. Informed consent was obtained, and the University Review Board approved the study, which was performed in accordance with the Helsinki Declaration. Five patients had hypertensive cerebral hemorrhage in the basal ganglia with a hematoma volume between 40 and 80 ml. All patients with ICH that was confirmed using computed tomography (CT) examination within 6 hours who required minimally invasive surgery within 12 hours under a microscope provided consent to undergo perihematomal sample collection. The five normal brain tissue samples were obtained from the non‐functional tissue in non‐stroke patients undergoing deep‐seated meningioma resection and used as controls. No significant differences in age or sex were found between the two groups.

### 2.2 Experimental study in rats

The Animal Care Committee of Zhengzhou University approved all procedures. Adult male Sprague Dawley rats weighing 275–300 g (provided by the Experimental Animal Center of Henan Province) were randomly assigned to four groups (total n = 265, see the supplemental material in Table 1): (A) sham operation, treated with needle insertion only; (B) ICH, a stereotactically guided, collagenase injection‐induced ICH model used to mimic right‐sided intrastriatal bleeding; (C) ICH + CAY10444, ICH model animals that received CAY10444 via intraperitoneal injection, freshly prepared CAY10444 dissolved in DMSO at a 1:1 ratio and diluted in PBS was administered once per day (0.5 mg^−1 ^kg^−1^ d^−1^) for 1 day or 3 days, and the initial CAY10444 therapy was administered 6 hours after ICH and subsequently administered every 24 hours beginning on the second day after ICH; and (D) ICH + vehicle, ICH animals that received vehicle administration, and an equal volume of PBS was administered using the same regimen. The rats were housed at 23 ± 2°C with a 12‐h light‐dark cycle and fed food and water ad libitum. All animals in our study were decapitated for sample collection under deep anesthesia. The brain tissues of rats were removed approximately 4 mm from the hematoma for subsequent experiments and stored immediately after death.

**TABLE 1:** The detail of rats sacrificed at multiple time points in different groups

Group	Time point (d)	Total RNA sequencing RT‐PCR	Neurological test brain edema MRI	Evans blue extravasation	Western Blotting Elisa	Immunofluorescence	Immunohistochemistry	Electron microscopy	Flow cytometry
Sham	1	3	6	6	6	—	—	—	3
	3	3	6	6	6	6	6	6	3
ICH	1	3	6	6	6	—	—	—	3
	3	3	6	6	6	6	6	6	3
ICH + CAY10444	1	—	6	6	6	—	—	—	3
	3	3	6	6	6	6	6	6	3
ICH + vehicle	1	—	6	6	6	—	—	—	3
	3	—	6	6	6	6	6	6	3

### 2.3 Experimental model establishment

The ICH model was established in rats as previously described.^18^ Briefly, collagenase VII‐S (sterile‐filtered, 0.5 U in 0.5 μL of sterile saline, Sigma, St. Louis, MO, USA) was injected into the right basal ganglia of rats at the following stereotactic coordinates: 0.2 mm anterior and 3.5 mm lateral to the bregma, and 5.5 mm in depth. No deaths occurred in the successfully modeled rats.

### 2.4 Cell lines and coculture

The human cerebral microvascular endothelial cell line hCMEC/D3 and a human astrocyte (HA) cell line were generous gifts from the Shenzhen Institutes of Advanced Technology, Chinese Academy of Sciences. A coculture model was developed in our previous study.^11^ Three to four days after the initial coculture, S1P (1 µM, Apexbio, USA) and CAY10444 (1 µM, Glpbio, USA) were added to the endothelial side.

### 2.5 Experimental model establishment

The ICH model was established in rats via the injection of collagenase VII‐S (sterile‐filtered, 0.5 U in 0.5 μL of sterile saline, Sigma, St. Louis, MO, USA) as previously described. Rats that died before the end of the study were excluded. Otherwise, all animals were included in the final analysis. The brain tissues of rats were removed approximately 4 mm from the hematoma for subsequent experiments and stored immediately after death.

### 2.6 Total RNA profiling analysis

Rat brain tissue on day 3 following ICH and human perihematomal brain tissue that was confirmed using CT within 12 h were used for RNA‐seq. RNA isolation and quantification were performed according to the protocols in previous reports,^11^ and sequencing of the total RNA profile was performed using a HiSeq 4000 (Illumina, USA).

### 2.7 Neurological score evaluation

ICH‐induced neurological deficits were assessed using forelimb placement, forelimb‐use asymmetry, and corner turn tests. Behavioral tests were performed as previously described.^19^, ^20^

### 2.8 Immunohistochemical staining

Immunohistochemical staining methods were described previously.^11^, ^21^ The samples were incubated with rabbit anti‐S1PR3 (1:100, Bioss, China), mouse anti‐NeuN (1:100, Bioss, China), mouse anti‐GFAP (1:100, Bioss, China), mouse anti‐CD31 (1:100, Abcam, USA), and mouse anti‐Iba‐1 (1:100, Bioss, China) antibodies (Abs). ZO‐1 quantitative colocalization analysis was performed in a previous study,^22^ and two independent observers performed the counts. The coverage coefficients of ZO‐1/CD31, S1PR3/CD31 were calculated using ImageJ.

### 2.9 Magnetic resonance imaging (MRI) examination

MRI was used to evaluate hematoma volume and brain edema 24 and 72 h after ICH. Rats were anesthetized with pentobarbital (45 mg/kg, i.p.) and placed in the prone position on a scanning bracket. The rat body temperature was maintained at 37°C using a heating pad. MRI was performed using a 3.0 T MRI scanner (uMR780, United Imaging, China). MRI studies were performed using a fast spin‐echo (FSE) sequence to calculate a T2 map. The following parameters were used during MRI acquisition: field of view (FOV) = 40 *40 mm, 1.5‐mm slice thickness, TR = 4,000 ms; TE = 92.4 ms; 240 × 240 matrix; bandwidth = 260, and number of excitations [NEX] = 4.

### 2.10 Brain water content examination and Evans blue (EB) analysis

The integrity of the BBB was investigated by measuring the extravasation of EB. The detailed procedures were described previously,^11^ and the results are shown as the ratio of ipsilateral (ipsi)/contralateral (contra) brain EB content. Brain water content was calculated as (wet weight−dry weight)/wet weight × 100%, and perihematomal edema was observed using MRI examination.

### 2.11 Quantitative reverse‐transcription PCR analysis (RT‐PCR)

RT‐PCR was performed according to a procedure based on a protocol described in a previous report.^20^ The following PCR primer sequences were used for target molecules: S1PR1: 5^/^‐GCTGAACATCGGAGTGGAGAAG‐3^/^(forward) and 5^/^‐GAGCCACAAACATACTTCCTTCC‐3^/^(reverse); S1PR2: 5^/^‐ATTTCTTTGCCTTCGCCACC‐3^/^(forward) and 5^/^‐CAGAAATGTTGGCGATGTAGGC‐3^/^(reverse); S1PR3: 5^/^‐TCATCGGCAACTTGGCTCTC‐3^/^(forward) and 5^/^‐CTACGAACATACTGCCCTCCCT‐3^/^(reverse); S1PR4: 5^/^‐CCTCTACTCCAAGGGCTATGTGC‐3^/^(forward) and 5^/^‐TTGGCTCGGACCACCCTAA‐3^/^(reverse); S1PR5: 5^/^‐GGCTAACTCGCTGCTGAATCC‐3^/^(forward) and 5^/^‐CTGTTGGAGGAGTCTTGGTTGC‐3^/^(reverse); and GAPDH: 5^/^‐CTGGAGAAACCTGCCAAGTATG‐3^/^(forward) and 5^/^‐GGTGGAAGAATGGGAGTTGCT‐3^/^(reverse). Relative changes in gene expression were quantified using the Livak method (2^−ΔΔCt^ method).

### 2.12 Flow cytometry detection

Spleens were mechanically processed in 2 mL of Cell Staining Buffer (BioLegend) over a 40‐mm strainer, and 30 µL was used for staining. Cell Staining Buffer was added to achieve a final volume of 100 µL. The following Abs (PE‐rat CD4, BioLegend, USA) in a total volume of 50 µL were added at the concentrations suggested by the manufacturer. Isotype control Abs were used at the same protein concentrations as the corresponding marker‐specific Abs. Cells were stained in the dark on ice for 20 min, and 1.6 mL of One‐Step Fix/Lyse Solution (eBioscience) was added, mixed, and incubated with the cells for 30 min at room temperature. The cell solution was centrifuged, and the supernatant was discarded. The cells were washed twice with Cell Staining Buffer, resuspended in 2 mL of Cell Staining Buffer, sorted on a FACSCanto II (Becton Dickinson Biosciences), and analyzed using Flow Jo V10.0.7.

### 2.13 Enzyme‐linked immunosorbent assay (ELISA)

Medium was collected from the upper chamber of a Transwell insert (Corning, USA) containing an hCMEC/D3 cell and HA coculture. Brain homogenates were prepared according to the manufacturer's instructions. Before measurement, brain tissue samples were collected 1 or 3 days after ICH. The levels of S1P (Ruixin, China), interleukin (IL)‐1β (eBioscience, China), tumor necrosis factor (TNF)‐α (eBioscience, China), and IL‐6 (eBioscience, China) were measured using commercial ELISA kits from eBioscience.

### 2.14 Western blot analysis

Rat brain tissue samples or cultured cells were prepared as descried in a previous report.^11^ Membranes were probed with primary Abs against CCL2 (ProteinTech 1:1,000, China), p‐p38 MAPK (1:1,000, Cell Signaling Technology, USA), ICAM‐1 (Bioss, 1:1,000, China), ZO‐1 (ProteinTech, 1:1,000, China), GAPDH (Abclonal, 1:10,000, China), and actin (Abclonal, 1:10,000, China) at 4°C overnight. The relative intensity of a protein signal was quantified via densitometric analysis using ImageJ software.

### 2.15 Transmission electron microscopy (TEM)

Briefly, the procedures for TEM examination were performed according to our previous report,^20^ and samples were observed using an electron microscope (JEM1440, Tokyo, Japan).

### 2.16 Statistical analysis

Genes were considered differentially expressed at a fold change ≥2 and *p* < 0.05. Parametric data are presented as the means ± standard deviation. The Shapiro‐Wilk test was used to assess the data distribution. One‐way analysis of variance (ANOVA) followed by LSD post hoc test were used to compare differences between multiple groups. Differences between two groups were evaluated using the two‐tailed Student's *t*‐test. All analyses were performed using GraphPad Prism 8.0 software. *p* < 0.05 was considered statistically significant.

## 3 RESULTS

### 3.1 Differentially expressed genes, including S1PR3 and CCL2, in rat and human samples

**FIGURE 1:**
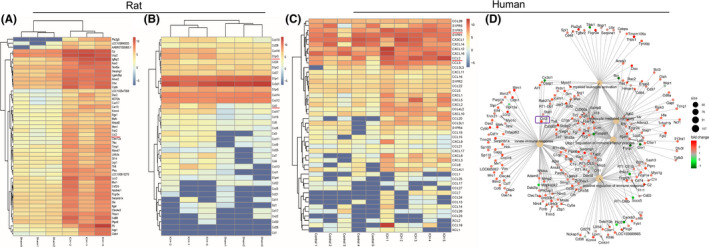
(A) Heat map of the fold changes in the top 50 differentially expressed RNAs in rats with ICH. The red line shows the CCL2 gene, ranked 25th. (B) Heat map of genes related to the immune inflammatory response in rats with ICH. The mRNA expression levels of S1PR3 (*p* < 0.01) in the ICH group were significantly higher than the sham group (the red line indicates rat S1PR3 and CCL2). (C) Heat map of genes related to the immune inflammatory response in patients with ICH. The mRNA expression levels of S1PR3 (*p* < 0.01) and CCL2 (*p* < 0.05) in the human ICH group were significantly higher than the control group (*n* = 3 rats, the red line indicates human S1PR3 and CCL2). (D) Functional network map indicating that CCL2 is a key molecule in the pathogenesis of the innate immune response and regulation of the immune effector process (the purple box indicates CCL2)[Colour figure can be viewed at wileyonlinelibrary.com]

High‐throughput RNA‐seq analysis revealed the top 50 upregulated genes after ICH in rats, and the elevated CCL2 gene in rats was ranked 25th (Figure 1A, see red line). Further investigation of potential genes related to immune inflammation following ICH revealed genes with significantly increased expression, including S1pr3 and Ccl2, in the rats in the ICH group compared with the sham group (Figure 1B, see red line). The differential gene expression of human specimens was also analyzed, and human S1PR3 and CCL2 expression levels were dramatically increased in ICH samples compared with normal samples (Figure 1C, see red line). A functional network map indicated that CCL2 was a key molecule in the pathogenesis of the innate immune response and regulation of the immune effector process (Figure 1D).

### 3.2 Upregulated S1P and S1PR3 expression and S1PR3 colocalization after ICH

**FIGURE 2:**
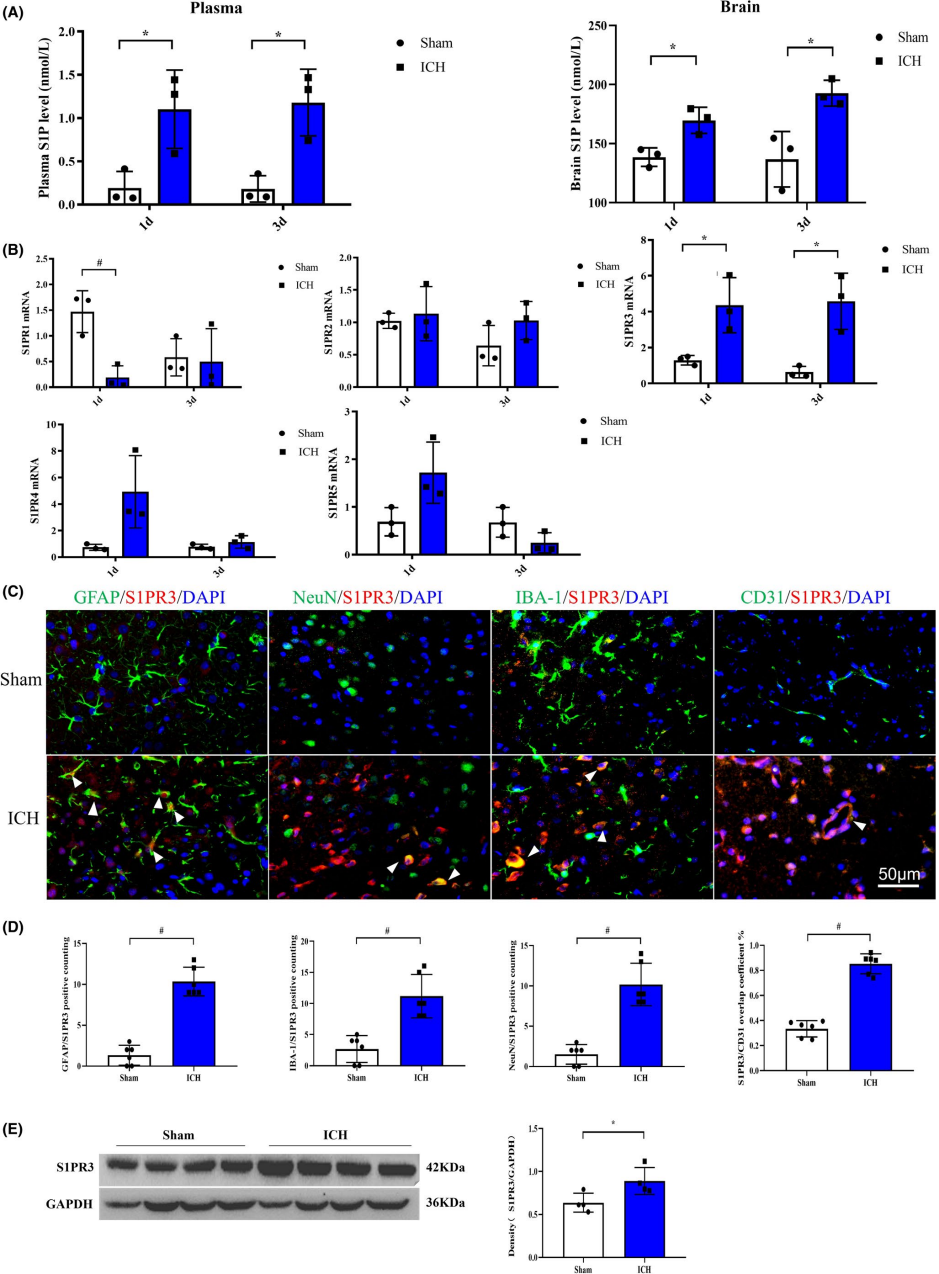
(A) ELISA showed that the concentration of S1P in the plasma was significantly increased on days 1 (*p* < 0.05) and 3 (*p* < 0.05) after ICH. The concentration of S1P in the brain was similarly significantly increased 1 day (*p* < 0.05) and 3 days (*p* < 0.05) after ICH (*n* = 3 rats). (B) RT‐PCR showed that the transcriptional level of S1PR1 mRNA was reduced, and the expression level of S1PR3 mRNA was markedly increased on days 1 and 3 after ICH compared with sham treatment (*n* = 3 rats,* indicates *p* < 0.05, # indicates *p* < 0.01). (C) Double immunofluorescence staining showed that S1PR3 colocalized with GFAP, NeuN, Iba‐1, and CD‐31, which suggests that S1PR3‐expressing cells originate from a wide range of sources, including astrocytes, neurons, microglia, and vascular endothelial cells. (D) S1PR3 colocalization with other cells increased markedly after ICH (*n* = 6 rats, # indicates *p* < 0.01). (E) The level of S1PR3 increased significantly after cerebral hemorrhage (*n* = 4 rats, *p* < 0.05)[Colour figure can be viewed at wileyonlinelibrary.com]

Our results showed that the expression of S1PR3 was dramatically increased after ICH in humans and rats. The concentrations of S1P (a ligand of S1PR3) in the serum and brain were evaluated. ELISA results demonstrated that the expression levels of S1P in the plasma and brain were significantly increased on days 1 and 3 in the ICH group compared with the sham group (Figure 2A). We further examined total S1PR subtype expression using RT‐PCR. RT‐PCR demonstrated that there were statistically significant differences in S1PR1 (1 day) and S1PR3 (1 day and 3 days) mRNA levels after ICH compared with sham treatment (*p* < 0.05). The mRNA expression of S1PR1 was downregulated, and S1PR3 was upregulated after ICH (Figure 2B). Double immunofluorescence staining revealed that S1PR3 colocalized with astrocytes, brain microvascular endothelial cells, neurons and microglia and a significantly increased number of co‐stained cells (Figure 2C,D), which suggested abundant production of S1PR3 by multiple cells after ICH. Western blot showed that the expression of S1PR3 increased following ICH (Figure 2E).

### 3.3 Reduced brain edema and EB extravasation and an improved BBB structure after S1PR3 inhibition

**FIGURE 3:**
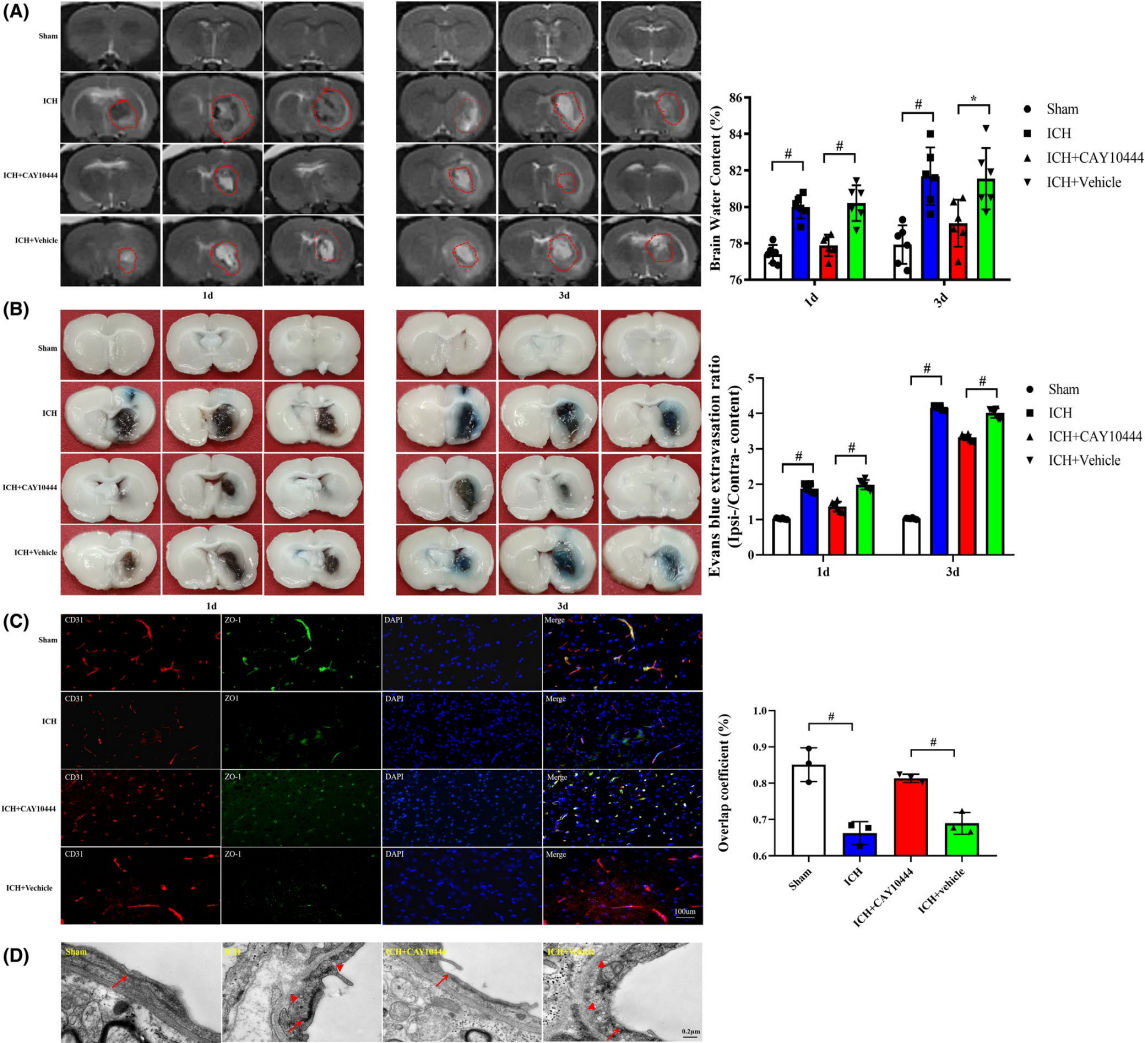
(A) T2‐weighted MRI showing the hematoma volume and perihematomal edema on days 1 and 3 after ICH in rats. The red dotted line indicates the size of the hematoma and the perihematomal edema. The histogram shows that the brain water content was significantly reduced in the CAY10444 group compared with the vehicle group on days 1 and 3 after ICH (*n* = 6 rats, * indicates *p* < 0.05, # indicates *p* < 0.01, white box = Sham, blue box = ICH, red box = ICH + CAY10444, green box = ICH + vehicle). (B) Representative morphologies of brain tissue samples demonstrating that EB extravasation occurred on days 1 and 3 after ICH. Diminished EB extravasation was observed following CAY10444 treatment compared with vehicle treatment on days 1 and 3 after ICH (*n* = 6 rats, # indicates *p* < 0.01, white box = Sham, blue box = ICH, red box = ICH + CAY10444, green box = ICH + vehicle). (C) Representative distribution of immunohistochemical staining revealed that the coverage of ZO‐1/CD31 was higher in the CAY10444 group than the vehicle group (Scale bars: 50 µm, *n* = 3 rats,* indicates *p* < 0.05, # indicates *p* < 0.01). (D) Typical TEM demonstrating that the vascular walls of the sham group were lined with regularly flattened endothelial cells and extensive tight junctions. BBB disruption resulted in capillary endothelial cell swelling (long arrows) and excessive vesicles (triangles) after ICH. However, improvements in the BBB, such as decreased vesicle numbers and restoration of tight junctions, were significant after CAY10444 treatment (Scale bars: 50 µm, *n* = 6 rats)[Colour figure can be viewed at wileyonlinelibrary.com]

To investigate the role of elevated S1PR3 expression, the brain water content, EB extravasation, and the BBB ultrastructure were examined. A representative image of alleviated brain edema acquired by T2‐weighted 3.0 T MRI is shown, and the brain water content was significantly reduced in the ICH + CAY10444 group compared with the ICH + vehicle group (Figure 3A). EB staining showed diminished EB leakage and ameliorated BBB function in the ICH + CAY10444 group compared with the ICH + vehicle group (Figure 3B). To further clarify the observed BBB amelioration, the ultrastructure of the BBB was revealed using TEM and immunostaining 3 days after ICH. Double immunofluorescence staining revealed that the overlap coefficient of ZO‐1^+^/CD31^+^ after ICH was lower than after sham treatment, and this trend was reversed when the ICH + CAY10444 group was compared with the ICH + vehicle group (Figure 3C). Representative morphologies observed on TEM showed that the basement membrane of the BBB was disrupted, and there were abundant vesicles on day 3 after ICH. The basement membrane of the BBB was partially repaired, and the vesicle abundance was reduced after CAY10444 administration (Figure 3D).

### 3.4 Inhibition of S1PR3 alleviates behavioral deficits and the inflammatory response

**FIGURE 4:**
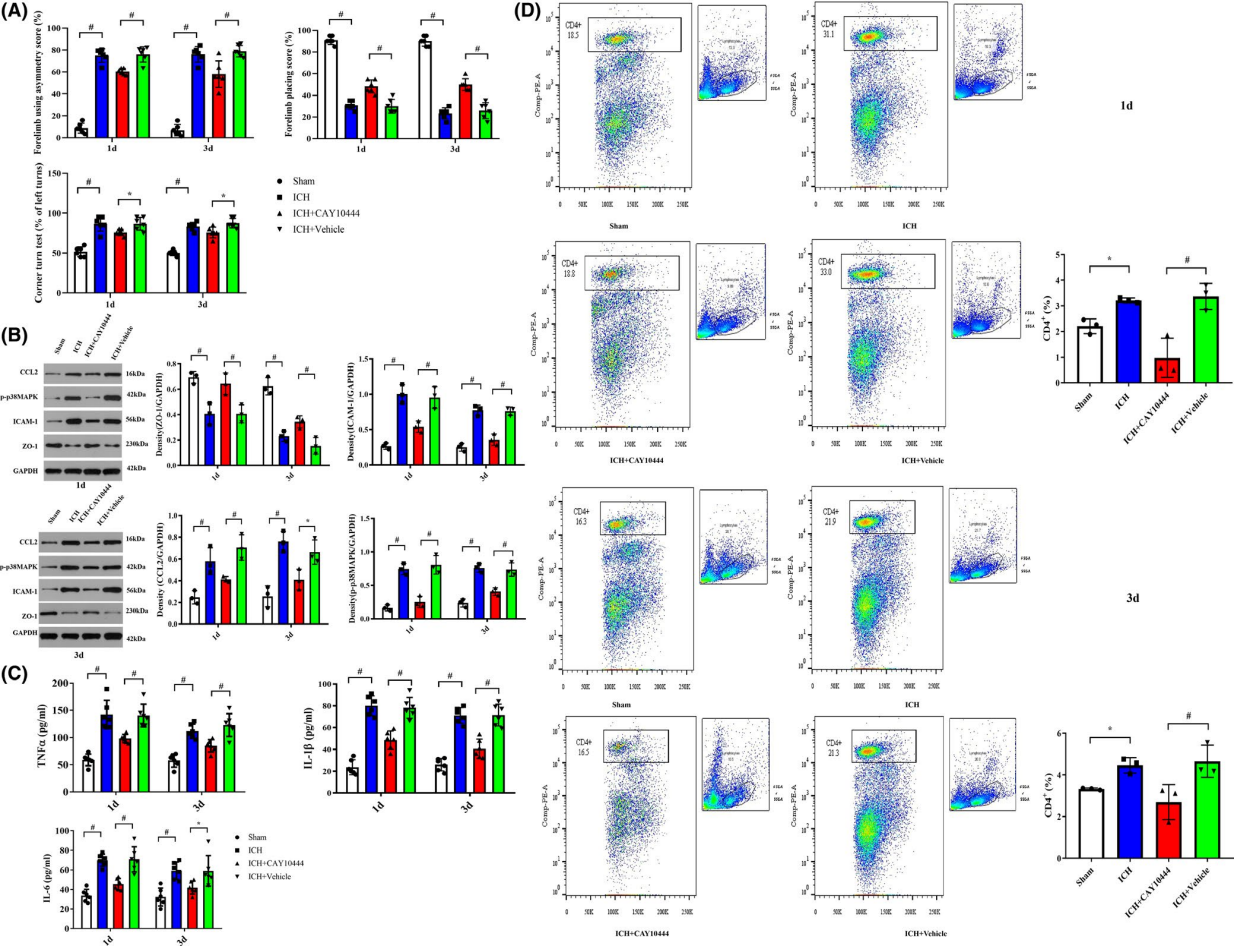
(A) Behavioral scores, including the forelimb placement score, corner turn test results and forelimb‐use asymmetry score, were significantly improved following CAY10444 treatment after ICH at 1 and 3 days (*n* = 6 rats, * indicates *p* < 0.05, # indicates *p* < 0.01). (B) Western blot analysis showed that CCL2, p‐p38 MAPK and ICAM‐1 expression levels were markedly increased in the perihematomal area on days 1 and 3 after ICH. ZO‐1 levels were decreased following ICH, and there were significant differences between the ICH and sham groups. However, decreased expression levels of CCL2, p‐p38 MAPK and ICAM‐1 were achieved after CAY10444 administration. A significant increase in the ZO‐1 level was observed in the ICH + CAY10444 group compared with the ICH + vehicle group (*n* = 3 rats, * indicates *p* < 0.05, # indicates *p* < 0.01, white box = Sham, blue box = ICH, red box = ICH + CAY10444, green box = ICH + vehicle). (C) The levels of cytokines, including IL‐1β, TNFα and IL‐6 (soluble microglial markers), were significantly upregulated in the perihematomal region after ICH. In contrast, obviously diminished IL‐1β, TNFα and IL‐6 expression levels were observed in the ICH + CAY10444 group compared with the ICH + vehicle group on days 1 and 3 (*n* = 6 rats per group. * indicates *p* < 0.05, # indicates *p* < 0.01). (D) Flow cytometry revealed that CAY‐10444, as an S1PR3 inhibitor, significantly inhibited the number of endotoxic CD4+ T cells in the spleen on days 1 and 3 after ICH in rats (*n* = 3 rats,* indicates *p* < 0.05)[Colour figure can be viewed at wileyonlinelibrary.com]

Behavioral scores, including the forelimb placement score, corner turn test result, and forelimb‐use asymmetry score, were significantly improved following CAY10444 treatment after ICH on days 1 and 3 (Figure 4A). To investigate the role of proinflammatory factors in ICH, we examined the production of inflammatory mediators, including IL‐1β, TNFα, and IL‐6, in rats after ICH. The levels of cytokines, including IL‐1β, TNFα, and IL‐6, were significantly upregulated after ICH. In contrast, obviously diminished cytokine expression levels were observed in the ICH + CAY10444 group compared with the ICH + vehicle group on days 1 and 3 (Figure 4C).

### 3.5 Suppression of S1PR3 affects proinflammatory factor expression and reduces the total quantity of CD4+ T cells in the spleen after ICH

S1PR3 inhibition exerted a neuroprotective function against secondary injury by preserving BBB integrity following ICH, and the mechanisms underlying this neuroprotection may be associated with decreased excessive inflammatory mediator expression and a reduced CD4+ T‐cell count. Western blot analysis showed that the CCL2, p‐p38MAPK and ICAM‐1 levels were significantly increased in rats with ICH on days 1 and 3, and the expression level of ZO‐1 was reduced after ICH compared with sham treatment. However, there was significantly decreased expression of CCL2, p‐p38MAPK, and ICAM‐1 and increased ZO‐1 expression after CAY10444 administration (Figure 4B). Flow cytometry revealed significant increases in the number of CD4+ T cells in the spleen on days 1 and 3 following ICH (*p* < 0.05), which were reversed by CAY10444 therapy for S1PR3 blockade (*p* < 0.05, Figure 4D).

### 3.6 S1PR3 blockade alleviates microglial activation, proliferation, and M1 polarization and promotes M2 polarization

**FIGURE 5:**
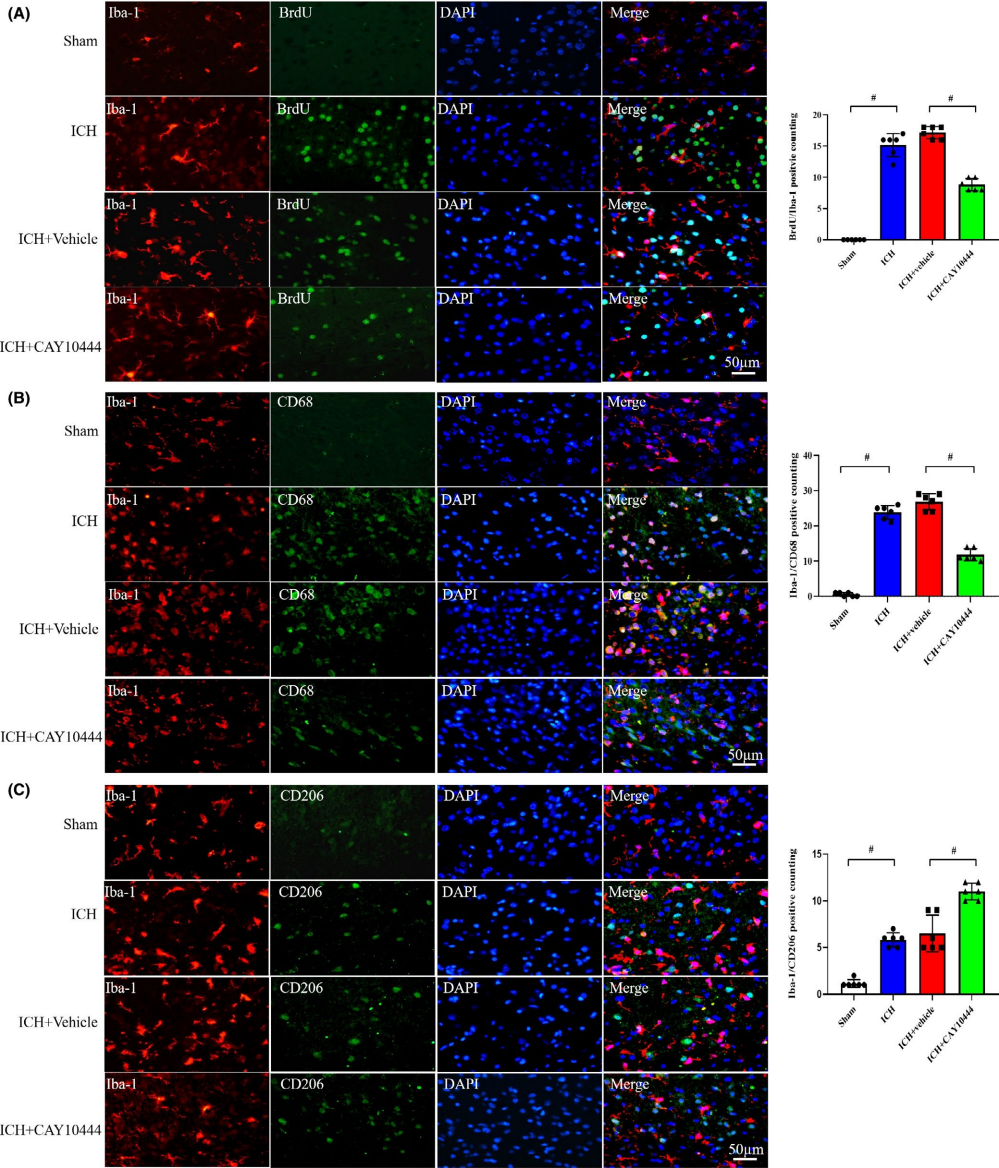
(A) Double immunofluorescence staining showed a significantly increased number of proliferating microglia following ICH (Sham versus ICH). However, amoeboid‐like microglia transformed to normal ramified morphology and microglial proliferation was attenuated after CAY10444 administration (Scale bars: 50 µm, *n* = 6, # indicates *p* < 0.01). (B) Representative image demonstrating decreased microglial activation and M1 microglial (Iba‐1+/CD68+) changes following treatment (Scale bars: 50 µm, *n* = 6, # indicates *p* < 0.01). (C) M2‐like microglial (Iba‐1+/CD206+) activation was increased following ICH and further CAY10444 administration (Scale bars: 50 µm, *n* = 6 rats, # indicates *p* < 0.01) [Colour figure can be viewed at wileyonlinelibrary.com]

The number of activated microglia was significantly increased in perihematomal issue (ICH vs. Sham), and BrdU/Iba‐1‐immunopositive cells were obviously reduced after CAY10444 administration (ICH + vehicle vs. ICH + CAY10444), which suggests that S1PR3 is involved in microglial activation after ICH (Figure 5A). CD68, which is a classic M1‐like (proinflammatory) phenotype of microglia/macrophages, and Iba‐1 were increased in ICH and showed significant decreases after S1PR3 blockade (Figure 5B). In contrast, the anti‐inflammatory M2‐like microglia polarization was promoted (Figure 5C).

### 3.7 S1PR3 and S1P modulate the CCL2‐p‐p38 MAPK pathway in vitro

**FIGURE 6:**
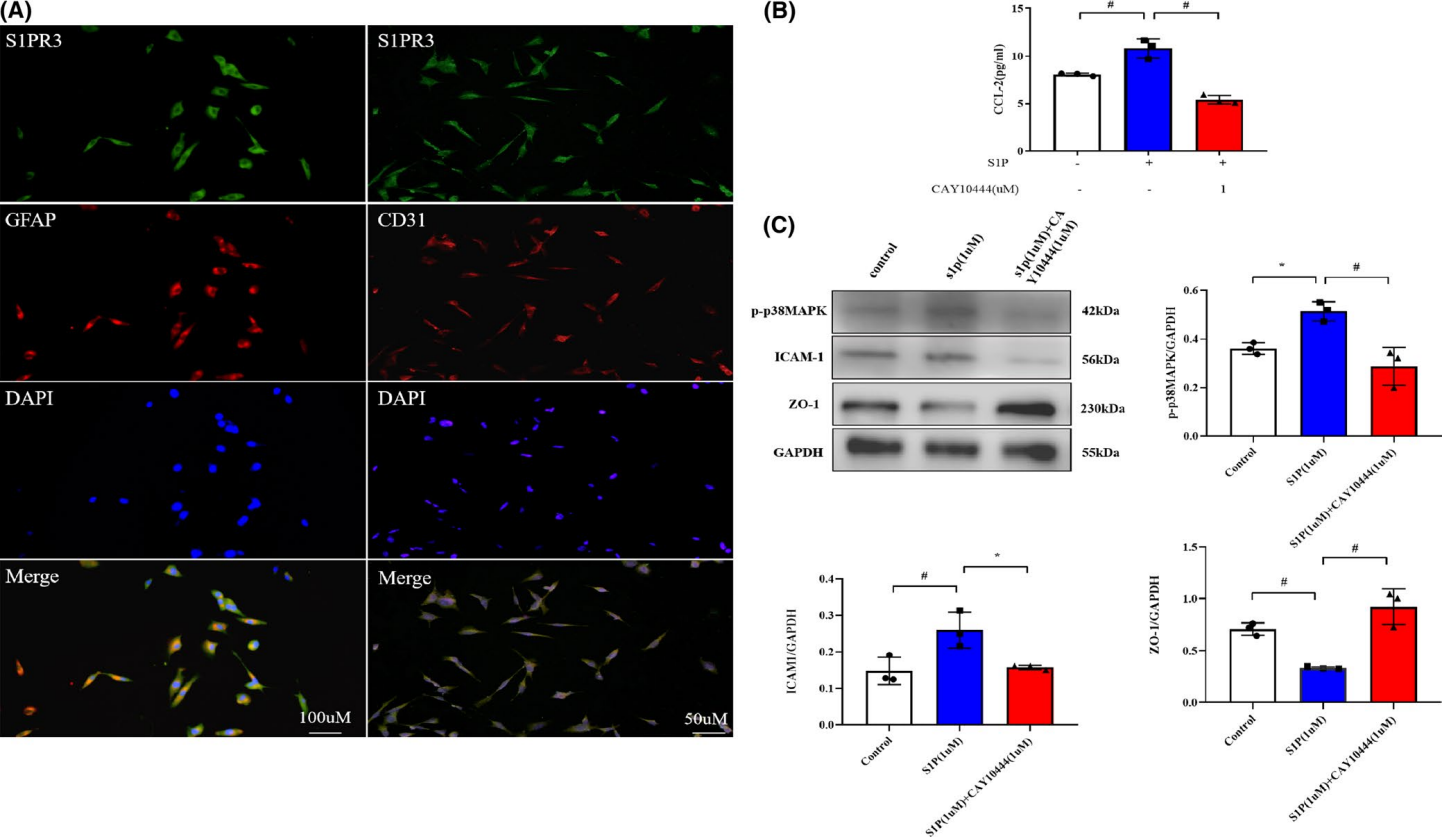
(A) In vitro double immunofluorescence staining showed that S1PR3 colocalized with GFAP and CD31, which suggests that S1PR3 is expressed on astrocytes and vascular endothelial cells, respectively. (B) ELISA revealed elevated CCL2 expression in HA +hCMEC/D3 cells after S1P stimulation (* indicates *p* < 0.05, # indicates *p* < 0.01). (C) Western blot analysis indicated that the expression levels of p‐p38MAPK and ICAM‐1 increased, and the expression of ZO‐1 decreased after S1P stimulation. However, the expression levels of p‐p38MAPK and ICAM‐1 were decreased, and the expression of ZO‐1 was increased in cocultures treated with S1P + CAY10444 (* indicates *p* < 0.05, # indicates *p* < 0.01) [Colour figure can be viewed at wileyonlinelibrary.com]

To analyze the potential regulatory mechanism of S1PR3 impact on the BBB, the expression levels of CCL2, p38 MAPK, and ICAM‐1 were analyzed in vitro. Double immunofluorescence staining revealed that S1PR3 colocalized with astrocytes and brain microvascular endothelial cells in culture (Figure 6A). With the addition of S1P (1 µM) to the hCMEC/D3 cell and HA coculture, CCL2, p‐p38 MAPK, and ICAM‐1 expression levels were increased, and there was a significant reduction in the ZO‐1 level at 24 h (Figure 6C). However, CAY10444 administration alleviated this phenomenon (Figure 6B), which indicates that S1PR3 inhibition exerts a neuroprotective effect via the S1P‐CCL2‐p‐p38 MAPK pathway.

## 4 DISCUSSION

The present findings suggest that S1PR3 plays a vital role in BBB disruption in rats with acute ICH. To the best of our knowledge, this report is the first study to demonstrate that CAY‐10444 exerted a neuroprotective effect via S1PR3 inhibition in a rat model of hemorrhagic stroke injury. The neuroprotection mediated by S1PR3 blockade was linked to the maintenance of BBB integrity via inhibition of excessive inflammatory and immunological response injury following ICH. The molecular mechanisms underlying the protective effects of S1PR3 inhibition in the context of ICH were associated with several factors: ① CCL2‐p‐p38MAPK signaling pathway activity was markedly attenuated; ② ICAM‐1, as a key immune cell recruitment factor, was inhibited after CAY‐10444 administration; ③ a marked decrease in the CD4+ T‐cell percentage in the spleen induced by immune suppression likely resulted in reduced immunological injury; ④ the levels of proinflammatory cytokines, including IL‐1β, TNFα, and IL‐6, were significantly diminished; ⑤ the number of proliferated microglia and M1 polarization decreased and M2‐like phenotype increased; and ⑥ the expression of the tight junction protein ZO‐1 was increased in vivo and in vitro.

Cartier A *et al*. noted that dysregulated S1P signaling disrupted the BBB, which is an early event that contributes to the pathogenesis of many CNS diseases.^12^ Prominent differential expression of diverse S1PR subtypes was found in the present research, and some S1PR subtypes, especially S1PR3, showed elevated expression, and the downregulation of molecules, such as S1PR1, occurred after ICH. The abundance of S1P and the dysregulated expression of S1PRs in perihematomal tissue pose key questions: What are the potential roles of S1PRs in BBB breakdown, and how do S1PRs regulate the downstream signaling axis? Recent studies revealed that the S1PR1 agonist RP101075, or BAF‐312, as a modulator of S1PR1/S1PR5, significantly attenuated neurological deficits and reduced brain edema in experimental ICH.^23^, ^24^ However, the overexpression of S1PR3 in our study played essential roles in BBB disruption and brain edema after ICH, and excellent outcomes, including improvements in neurological deficits and BBB integrity, were obtained after CAY‐10444 administration. It would be significant and helpful to intensively investigate the multifaceted possibilities of focusing on S1PR as a novel target for the development of therapeutic paradigms and the discovery of new drugs for ICH in the future.

Growing evidence showed that ICH produces local damage to brain and causes systematic inflammation and immune dysregulation.^25^, ^26^, ^27^, ^28^, ^29^, ^30^ A modest inflammatory response promotes neurogenesis, but an excessive inflammatory cascade aggravates secondary brain injury, especially at an early stage, which is primarily achieved via proinflammatory cytokines, leukocyte infiltration, and activated microglia. M1‐like microglia exhibit a proinflammatory response as early as 6 h after ICH, and an M2 phenotype contribute to hematoma resolution and neural repair.^28^, ^31^, ^32^ Therefore, the prevention of neurotoxicity targeting early M1‐like microglial response has a considerable benefit. An increasing number of recent research focused on the modulation of microglia polarization to ameliorate brain injury after ICH.^33^, ^34^ We hypothesis that a better prognosis may be achieved by reducing the number of M1 microglia and controlling the shift from the M1 to the M2 phenotype via S1PR3 inhibition. Notably, some researchers reported that the suppression of S1PR3 did not alter the expression of M2 markers, which suggests that it primarily exerts its neuroprotective effect via decreased proliferation and an M1‐like response.^35^ However, significant increases in an M2‐like phenotype were observed in our study, which also demonstrated a crucial anti‐inflammatory role post‐ICH. Our study found that the levels of proinflammatory cytokines, including TNF‐α, IL‐1β, and IL‐6, were significantly reduced by CAY10444 administration after ICH. The sympathetic nervous system/hypothalamus‐pituitary‐adrenal axis is likely the first mode of communication between the CNS and peripheral immune system, and it mediates splenic shrinkage.^25^, ^30^ Splenectomy decreased monocyte/macrophage infiltration into the ischemic brain and alleviated brain injury, which suggests these spleen‐derived monocytes play an important role in ischemic stroke brain injury.^36^ Significantly decreased total numbers of CD4+ T cells were detected in the spleen, which led to a reduction of circulating lymphocytes after CAY10444 therapy. S1PR3 blockade led to an improved prognosis in the present study and may be associated with the decrease in brain‐infiltrated CD4+ T‐cell numbers,^29^ which resulted in less immunological inflammatory damage and diminished proinflammatory cytokine production. Recent studies showed that PD‐L1 treatment reduced the percentages of brain‐infiltrating CD4+ T cells, Th1 cells, and Th17 cells and increased the percentages of Th2 and regulatory T cells (Tregs), which reduced the inflammatory response after ICH.^37^ Another study showed that intravenous tail vein injection of Tregs significantly reduced brain water content and ameliorated short‐ and long‐term neurological deficits.^38^, ^39^ Considering the critical role of the immune response in secondary brain injury, early intervention targeting the excessive inflammatory response via immune regulation may be an effective approach for ICH.^40^

Our previous research confirmed that the chemokine CCL2 was a key inflammatory factor that contributed to BBB breakdown after ICH. CCL2 is one of the most potent microglial chemoattractants, and it plays vital roles in the regulation of the movement of microglia and their recruitment to sites of inflammation.^41^, ^42^ Notably, excessive S1P and S1PR3 expression and elevated CCL2 expression were also found after ICH in the present study. Because S1P induced CCL2 mRNA expression, as indicated in previous literature,^43^ we hypothesized that the increased expression of CCL2 would result from S1P and S1PR3 signal transduction. Reductions in brain edema content, amelioration of BBB integrity, and improvements in behavioral deficits were significant after the administration of an S1PR3 antagonist, CAY10444, following ICH in our study. The potential mechanisms for maintaining BBB integrity may be associated with inhibition of the S1PR3‐CCL2‐p38MAPK signaling pathway. The CCL2 and p‐38MAPK levels were diminished after S1PR3 inhibition in vivo, and the expression of CCL2 and p‐p38 MAPK was significantly decreased after S1P stimulation plus S1PR3 antagonism in vitro. Accumulating evidence demonstrated that adhesion was mediated by interactions between β2‐integrins on leukocytes and ICAM‐1 on cerebral endothelial cells, and FTY720, which modulates S1PR, decreased ICAM‐1 expression, which contributes to the amelioration of leukocyte plugging.^35^ Our results suggest that the neuroprotective role of S1PR3 inhibition after ICH may be linked to decreased ICAM‐1 levels and reduced proinflammatory CCL2 and p‐p38 MAPK expression. Taken together, these factors may explain the underlying mechanism (see Figure S1).

## 5 CONCLUSIONS

In summary, the present study revealed a novel role for S1PR3 in modulating the CCL2 pathway after ICH. The potential molecular mechanisms of protection mediated by S1PR3 inhibition may be involved in the immunomodulation of early inflammatory responses and enhancement of BBB integrity. To the best of our knowledge, this report is the first study to demonstrate the efficacy of CAY10444 in hemorrhagic stroke, and targeting S1PR may be a promising therapeutic treatment in the future.

## CONFLICTS OF INTEREST

The authors declare no competing interests.

## AUTHORS’ CONTRIBUTIONS

Guo FY, Xu DK, and Gao Q designed the research. Guo FY, Xu DK, Gao Q, Wang F, and Peng QR performed the research and data analyses. Wang GQ, Wei QJ, Lei SX, Zhao SQ, and Zhang LX performed basic studies. Guo FY and Xu DK wrote the paper, and Guo FY critically revised the paper.

## ETHICAL APPROVAL

The Ethics Committee for Human Experiments of Zhengzhou University approved all procedures, and all animal handling and surgical procedures were performed in accordance with the approved animal protocols, as specified by the Institutional Animal Care and Use Committee at Zhengzhou University.

## Supporting information

Fig S1

## Data Availability

The datasets used and/or analyzed during the current study are available from the corresponding author on reasonable request.
