# Supplementary figures and images for: Sphingosine‐1‐phosphate receptor 3 is implicated in BBB injury via the CCL2‐CCR2 axis following acute intracerebral hemorrhage
Source: CNS Neurosci Ther. 2021 Feb 28;27(6):674–86. doi: 10.1111/cns.13626 (PMC8111497; doi:10.1111/cns.13626)

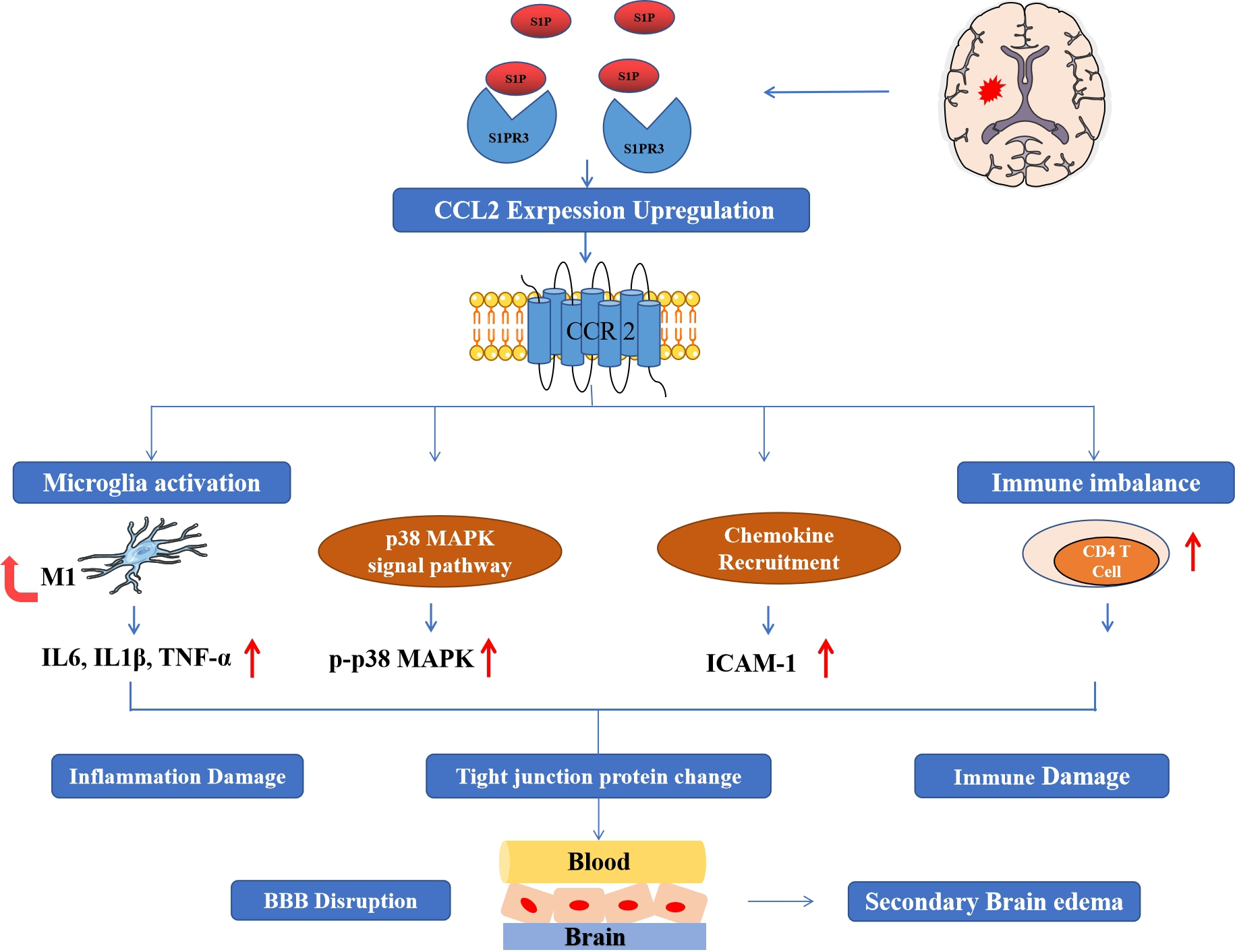

Supplement: Supplementary file 1 — Fig S1 [file CNS-27-674-s001.tif]
